# An exploration into the occupational identity of women following breast cancer and treatment: A qualitative study

**DOI:** 10.1177/03080226231225103

**Published:** 2024-01-09

**Authors:** Courtney Hopkins, Angela Murphy, Rebecca Haythorne, Daniel Cezar da Cruz

**Affiliations:** School of Health, Leeds Beckett University, Leeds, UK

**Keywords:** Breast cancer, occupational identity, occupational participation, oncology, engagement

## Abstract

**Introduction:**

The number of women surviving after breast cancer is increasing, along with the length of time they are living with the after-effects of treatment. Although the treatment’s effects are known to impact occupational participation, little is known about how breast cancer could affect occupational identity. This study aims to illuminate the lived experience of women long-term after breast cancer treatment through an occupational perspective in order to explore how they perceive their occupational identity.

**Methods:**

A qualitative study with semi-structured interviews was conducted with six women, who had all received a diagnosis of breast cancer and treatment for longer than a year. Reflexive Thematic Analysis was used to analyse the data.

**Findings:**

Three intertwined themes describe the participants’ experience. (1) ‘Disruptions in daily life and Environmental support’, (2) ‘Be able to do’ and identity, and (3) ‘Doing what matters and is possible’. Findings revealed that the occupational identities of the participants were maintained. Cancer treatment effects appear to impact occupational competence that corresponded to participants’ occupational identities, suggesting difficulties in the order of occupational adaptation.

**Conclusion:**

Our findings contribute to understanding the challenges to occupational participation related to the occupational identity of women following breast cancer and treatment.

## Introduction

In 2020, it was reported by The World Health Organization (WHO) that since 2015, 7.8 million women have been diagnosed as living with breast cancer (WHO, 2021). Cancer Research UK (2020) reports that the survival rates for living with breast cancer for 5 and 10 years have increased to 85% and 75.9%, respectively. This means a growing number of this population is living with the effects of cancer and its treatments for longer (Johnson, 2015).

The primary forms of treatment for breast cancer are surgery, chemotherapy, radiotherapy, hormone therapy and targeted therapy (Jakobsen et al., 2018; Thomsen et al, 2023). Side effects of treatment can include cancer-related cognitive, fatigue and upper-extremity impairments, lymphedema and chemotherapy-induced peripheral neuropathy (Baxter et al., 2017; McGrath, 2013; Pergolotti et al., 2016). These side effects have been found to impact on occupational participation in meaningful occupations and possibly women’s occupational identity (Fangel et al., 2013; Fleischer and Howell, 2017; Jakobsen et al., 2018; Keesing et al., 2018; Thomsen et al, 2023). Despite the critical relationship between occupations, health and well-being already being established (Wilcock, 2007), occupational therapy is not usually provided once a person is discharged from acute services or until the person is at the end-of-life phase (Johnson, 2015), because cancer has been considered a chronic condition due to the lasting effects caused by the disease itself and its subsequent treatments (Baxter et al., 2017).

The occupational disruption caused by chronic illness has been documented to impact occupational identity (Nizzero Cote and Cramm, 2017). Occupational identity is a central concept to understanding humans as occupational beings (Hansson et al., 2022). Based on the seminal work by Christiansen (1999), who asserted that occupation is key to creating and maintaining an identity, Kielhofner (2008) proposed occupational identity in the Model of Human Occupation (MOHO) as: ‘a composite of self of who one is and wishes to become as an occupational being generated from one’s history of occupational participation’ (p. 106). In MOHO, occupational identity is a concept that evolves according to the individual’s participation in occupational roles (Bowyer et al., 2024), while occupational competence refers to how participation manifests a person’s identity (Kielhofner, 2008). Both occupational identity and competence occur within the environment. The interaction between these elements will inform a person’s occupational adaptation. In other words, occupational adaptation results when occupational identity matches the competence of the person within the environment (Bowyer et al., 2024; Kielhofner, 2008).

## Literature review

There is evidence reporting the impacts of breast cancer and its treatment on women’s daily lives. Experiencing side effects of breast cancer treatment has been found to cause feelings of dissatisfaction with participation in meaningful occupations, which can last several years post-treatment (Brick et al., 2021). In a recent longitudinal study conducted in Israel, Loubani et al. (2002) compared women’s participation in the subacute (2 years post-diagnosis) and chronic phases (5 years after diagnosis) to identify factors associated with participation and the strategies to cope with restrictions on participation. They found that lower levels of participation were related to higher symptom severity of cancer treatment. The authors suggest a need for a comprehensive assessment and early occupational therapy intervention since breast cancer-related symptoms were found to impact participation 5 years after breast cancer and its treatment. In addition, Thomsen et al. (2023) conducted a qualitative study with seven Danish women after breast cancer. Using two qualitative focus groups, they investigated engaging in football fitness as a meaningful driver of occupational identity. The main findings revealed that participants strengthened social relationships (connection with other breast cancer survivors) that allowed them to share and learn through common experiences, increasing motivation to change their lifestyle to engage in fitness, a sense of competence and unity as a group.

Research into the effects of breast cancer and its treatment has found issues related to participation in occupations that are purposeful and meaningful and that activity levels do not return to previous levels for months or even years after treatment completion (Fleischer and Howell, 2017). Nevertheless, little is known about the factors preventing the return to pre-diagnosis levels or how this population manages their occupational difficulties (Fleischer and Howell, 2017; Loubani et al., 2002). McGrath (2013) found that occupational therapy is not provided to this population to support participation in meaningful occupations further and that this occupational loss can cause a change in identity, occupational roles and well-being, with some individuals ignoring medical advice to maintain these aspects of their occupational lives. Research conducted by Fangel et al. (2013) reported that dependency on others can result in the loss of occupational identity due to not participating in occupational roles, and it can impact the quality of life of cancer survivors.

Despite the increase in and length of survival rates, studies focused on the occupational identity of women after breast cancer and treatment are limited. Our study, therefore, aims to gain insight into the lived experience of women long-term after breast cancer treatment through an occupational perspective in order to explore how they perceive their occupational identity over time. To respond to this aim, a research question was formulated: How is the perception of occupational identity described by women living after breast cancer and treatment, and what are the long-term effects of this experience in their lives?

## Methodology

### Study design

To explore the subject of occupational identity amongst women after breast cancer treatment, this study used a qualitative approach to capture the participants’ subjective experience (McGrath, 2013; Molitor et al., 2023). A qualitative, hermeneutic phenomenological approach was used to explore and gain insight into the participants’ experiences over time (Creswell and Poth, 2018; Van Manen, 2016), including how life has changed since diagnosis (Johnson and Christensen, 2019; Phelan and Kinsella, 2014). Semi-structured interviews with open questions were utilised to explore the lived experience of participants in the specific context of their lives (Creswell and Poth, 2018).

### Ethics

Ethical approval was granted via the Local Research Ethics Coordinator at Leeds Beckett University. Due to the nature of the topic, the interview schedule was provided to the participants before they gave their informed consent by returning the completed consent form. This allowed the participants to begin thinking about their answers and deciding if there were any questions they did not want to be asked, mitigating the potential for distress to be caused. All participants were allocated pseudonyms to uphold confidentiality, and any identifiable information was removed.

### Participants and recruitment

Criterion purposeful sampling was employed as women volunteering to participate had to have received a diagnosis of breast cancer longer than a year ago, not be under the care of occupational therapy, have no other condition and be over 18. Participants responded to the recruitment advert posted on the researcher’s professional Facebook and Twitter accounts. The charity ‘Breast Cancer Now’ also created an advert for the research, which was featured on their website. Six participants responded to the adverts (three via Breast Cancer Now and three via Facebook) with no dropouts. Basic information on the researcher’s identity (an MSc Occupational Therapy – Pre-registration – student) was clear in the participant information form as well as the aim of the research.

Since the aim of the study was to explore the lived experience of participants, we did not focus in depth on details of variables such as breast cancer characteristics, cancer treatment and demographics of participants; instead, we wanted to know their experience regarding occupational identity, congruent with the studies of Palmadottir (2009) and McGrath (2013). Moreover, recent evidence did not identify correlations between those characteristics and participation in meaningful occupation in women after breast cancer in their chronic phase; 5 years after treatment (Loubani et al., 2022).

All six participants were White British. Four were single, and two were married. They worked in healthcare, education and offices. The average years since diagnosis was 12.3 years, ranging from 2 to 20 years. Treatments reported included surgery and chemotherapy, mastectomy, chemotherapy and radiotherapy, radiotherapy and surgery, chemotherapy, surgery, radiotherapy and chemotherapy. Three participants reported cancer recurrence: secondary breast cancer, lung and triple-negative breast cancer.

### Instrument

Semi-structured interviews were selected as they allowed follow-up probes to elicit further detailed information and understand further participants’ lived experiences and the associated meanings (Adeoye-Oltunde and Olenik, 2021). The semi-structured interview was designed to capture the participant’s occupational history since, according to Kielhofner (2008), occupational identity comprises a ‘sense of who one is and wishes to become as an occupational being generated from one’s history of occupational participation’ (p. 109). Table 1 presents the interview questions and prompts.

**Table 1.** Interview questions and prompts.

Questions	Description
1.	What occupations did you carry out on a typical day before your diagnosis and treatment?
2	What occupations did you carry out on a typical day after your diagnosis and treatment?
3.	What was your experience of your diagnosis and treatment? Can you describe to me what impact this had on your typical occupations?
4.	How was your typical weekend during your diagnosis and treatment?
5.	What is your experience now, after the diagnosis and treatment? Can you tell me about your typical weekend? Prompts: Can you tell me a little more about that? What did that occupation involve? Can you explain that in a little more detail? How did you prepare for that? You mentioned [. . .]. Can you explain that in more detail? Can you tell me/explain to me why you chose that occupation/why you did it? How did participating in this occupation make you feel? Can you describe this feeling in more detail? On some occasions, you have mentioned [. . .]. Is that important to you? Can you explain why? Do you feel this occupation impacted how you viewed yourself/your identity? Can you explain that a little bit more? Can you describe why you stopped/started this occupation? What was meaningful about that experience for you?

### Data collection and data analysis

Participants contacted the principal researcher (CH) directly, and any questions and queries were answered via email before the interviews took place. There was no pilot test of the interview schedule. The interviews were conducted via either the telephone, Zoom or Microsoft Teams, depending on the participant’s preference. The interviews lasted between 30–45 min. The interviews were audio and, when applicable, video recorded. Field notes were completed throughout the research process before and after each interview. The interviews were transcribed verbatim onto Microsoft Word.

Reflexive Thematic Analysis (RTA) was chosen as it has been argued that it is a valuable method to highlight similarities and differences between individuals’ experiences (Nowell et al., 2017). The data were analysed thematically, following the six steps set out by Braun and Clarke (2021): familiarise with data (reading and re-reading), coding, generate initial themes, develop themes, refine themes, and write up. Three themes overlapped through this iterative process.

Reflexive logs and discussions between the principal researcher (CH) and the last author (DC) were employed to ensure reflexivity and confirmability, compatible with an interpretive hermeneutic approach. The analysis was inductive, deductive as well as latent, and semantic since RTA is flexible and does not require an option for one or another (Braun and Clarke, 2021; Byrne, 2022). The semantic analysis considered what participants said objectively, while the latent content was interpreted by the authors. The deductive analysis comprised the occupational terminology to create codes, themes and further discussion of the findings.

### Rigour and bias

Audio/video recording of the interviews allowed the researcher to ensure high levels of credibility by allowing them to revisit the interviews and ensure that the interpretation truly reflected the data (Nowell et al., 2017). As noted above, the interviews were transcribed and coded by the principal researcher (CH), and the themes and codes were discussed with the fourth author (DC) on Microsoft Teams to check and redefine codes and themes (Nowell et al., 2017). A third round of revisions was conducted together with all authors, who reviewed the final themes. Criterion purposeful sample ensured the trustworthiness of the data as this meant the participants would have expert knowledge through their lived experience (Adeoye-Olatunde and Olenik, 2021). Potential researcher influences were considered (Creswell and Poth, 2018) recognising the positionality and background of researchers ‘shaping interpretation’ (p. 34). To reduce bias, we can argue reflexively that although the first, second and third authors were females and this could be seen as bias due to their lived experience as women, the fourth author was a male, contributing to the analyses and discussion from a different perspective. None of the authors had experienced a breast cancer diagnosis. However, they had relatives or friends who had experienced breast cancer. Finally, the article followed the Consolidated criteria for reporting qualitative research to report the study (Tong et al., 2007).

## Findings

Three intertwined Themes describe the participants’ experience: ‘Theme 1: Disruptions in daily life and Environmental support’, Theme 2: ‘Be able to do’ and identity, and ‘Theme 3: Doing what matters and is possible’. Each theme presents verbatim quotes from the participants. This approach reflected the nature of understanding participants’ unique experiences rather than generalising the meaning of their experiences. Still, it allowed a level of interpretation that remained grounded in the participants’ perspectives.

### Theme 1: Disruptions in daily life and environmental support

Participants spoke about the various side effects of cancer and the impact these had on their daily life, including fatigue that in some cases contributed to a disruption of their desired activities such as work, leisure and social interactions:

Like a zombie, and I don’t mean the active zombies, I mean, the original zombies’ due to the fatigue . . . suffered after treatment (May).

I just feel like a waste of space. Sometimes I just I feel like I can’t keep up with other people and because I can’t do the things, I used to be able’ [. . .] really struggling to cope at school because of my breathing difficulties and my voice, I couldn’t. . .I didn’t have the voice projection that I had before (Hope).

The lived experience of one participant showed how discontinuing the ability to work due to surgery represented a sign of weakness to her:

I was signed off work around the time of the surgery, and that was really hard. I think I. . . type of person that, you know, didn’t want to admit weakness (Rachel).

Another participant considered the importance of waiting for the right time to overcome the effects of treatment to participate in dressing and the meaning of this activity associated with her identity as a woman:

Then the other thing is sort of clothes and being able to sort of feel like a woman, it’s almost like you once you’ve had surgery and radiotherapy for your breast to get back to normal takes quite a long time. . . . So there’s a bit coming to terms with that, being a woman and being feminine and dressing up (Rachel).

Participants shared their lived experiences facing barriers and how support from others and technology, on the one hand, enabled them to participate, and in specific situations that restricted their participation.

. . .first lot of chemo where I used the wheelchair quite a bit. Because my legs weren’t working and my fatigue was bad and I didn’t really like that, but it was a necessity. . . (Hope).

Because I wouldn’t be able to do the stairs and steps in the theatre, and especially because she had planned the seats by then and we were in the middle of a row. . . I couldn’t take the wheelchair and stuff (Elizabeth).

Social support from others appears to be an adaptation to sustain participation:

I couldn’t do embroidery because I couldn’t feel a needle, couldn’t thread the needle. . . I could still cook but my husband had to chop the vegetables, I couldn’t hold a knife properly’ (May).

My mum used to come up when I was under my chemo cycle. She used to cook. Do the washing and help me with the boys because it knocked me out (Hope).

### Theme 2: ‘Be able to do’ and identity

Although the environmental support was reported/perceived as enabling sustained participation for some participants, the lived experience revealed that the effects of not being able to participate independently had consequences perceived, for example, avoiding some social groups, feeling disappointed or resulting in mental health issues for not being able to have their skills to enact what they wanted to do:

I would have gone up to my mums for a meal, rather than having people at my house for dinner . . .it’s like it’s nice to repay people if they’re doing it for you as well and you feel a bit useless (Josephine).

I’d see things around the house and think, I can’t do that. And when other people are doing them for you, they don’t do it like you want to, so it was frustrating (Elizabeth).

I used to cook a roast dinner on a Sunday and it’s my pride that I’ve managed to keep doing the Sunday dinner, but at that point in time I could barely stand to cook anything. . . someone would have to help me or take over or do part of it, or whatever, so that had a detrimental effect on my mental health (Hope).

One participant described how not being able to continue going to work affected part of who she was and having an involvement with her previous occupation helped her to continue participating:

. . .Sense of bereavement really, bit like I lost a part of myself. . . because I’d lost my vocation (Hope). I was just like a helper in the class in a way, but they used to treat me as though I still worked there. . . so that helped (Hope).

Three participants expressed their frustration associated with breast cancer and its impacts on what they could do, conflicting with their identities and the way they viewed themselves, as Anna described the meaning of someone doing nothing by using the term ‘couch potato’:

I found it very frustrating because I didn’t have the physical energy or the physical strength to do anything that comes from being a really active presence, I became a couch potato and I really struggled with that (Anna).

The lived experience of participants also shows their unique perspectives. Hope described her feelings of being disabled due to the effects of treatment on her ability to do things, possibly assuming an identity as disabled, while May and Rachel refused to identify themselves by a diagnosis and its consequences:

I’m registered disabled now. And I feel disabled really, by the way the treatments affected me (Hope).

What I don’t want is to get the identity, Oh. May, with breast cancer (May).

Maybe for people that work in health care, there’s an extra challenge because you’re suddenly the patient. . . trying to marry the two things that you, your identity is you, but you’re also a survivor of breast cancer. . . it’s almost like I hadn’t really accepted I’d have breast cancer (Rachel).

### Theme 3: Doing what matters and is possible

Overall, participants shared their lived experiences seeking possible things to do. In particular, doing things for themselves and with others. Rachel expressed the importance of participating in adapted yoga routines during her treatment, affecting her physical and mental health positively:

I would say, from a physical point of view, feeling the strength of your body at a time when you’re feeling quite broken is really helpful. . . and you know, for my psychological profile, it helped me to get out of my head (Rachel).

Rachel also described the importance of going to visit Cornwall (UK) with her friend during her treatment, upholding her role as a friend and how being present in the moment could help to be away from the diagnosis or identity of a patient who receives treatment:

I was on the beach . . .and so remembering there’s another side of you, you know, you’re not just a patient, you know, a friend, you know, like, I love to go exploring and see new things. So that really, really helped [. . .] Connecting with the environment and connecting with the fact the world isn’t your diagnosis and your treatment. . . if the weather was nice, you know you could feel the sun on your face. You can bring yourself into that present moment (Rachel).

On the other hand, the lived experience after breast cancer allowed participants to know themselves in terms of what they could do and how to adapt to their occupations. Hope and Josephine revealed their strategies for pacing, doing only what was possible, with times for resting.

I’ve seen over the last few years the importance of pacing myself and not doing more than. . .doing things with resting (Hope).

If I was tired, I would just go back to bed and then I’d get up and do my cleaning, which would take longer, then I’d go back to bed again. I had to intersperse everything with a little rest or a little nap (Josephine).

A balance between challenges and skills can be identified in Rachel’s discourse, where she reported the need to adapt what she could do, requiring less physicality and at the same time providing comfort and entertainment, for example, returning to a previous occupation of reading a book:

I found that I just needed comfort reading, like maybe books I’ve read before, nothing too challenging. I remember, . . . I’d try to start a new book and I remember sitting in the waiting room for an appointment, and I just thought, Oh, this is too much like hard work. . . I had Harry Potter on my Kindle, you know, so it was almost like, you’re looking for something familiar and comforting. But also entertaining rather than something that’s going to sort of stretch you in any way (Rachel).

Hope explained how, despite the side effects of her treatment, she still participated in crafting and how doing the activity for hours was seen by her as entertainment that shifted the focus on her pain:

I . . . still do crafts, but it’s not as comfortable doing them and I can’t do it for so long. I need to do that because it’s a leisure type thing, it helps me to switch off from the pain (Hope).

## Discussion

Our study aimed to explore how women with breast cancer perceive their occupational identity and its long-term effects after cancer treatment. The main findings will be discussed deductively using an occupational perspective by interpreting participants’ experiences with an ‘occupational lens’- focusing on what people do in their everyday lives as occupational beings (Christiansen and Haertl, 2024; Njelesani et al., 2014).

The concept of occupational adaptation underpins our discussion. According to Grajo et al. (2018), there are two main theories of occupational adaptation: the MOHO and the Model of Occupational Adaptation. MOHO theory was chosen since it is coherent with the research conceptualisation of occupational identity as a component of occupational adaptation. Thus, occupational adaptation is a process and outcome impacted by the environment. As a process, occupational adaptation comprises the occupational identity (who a person is), the occupational competence (how a person sustains the occupational participation that corresponds to their identity) in interaction and support with the environment (Bowyer et al., 2024). As an outcome, occupational adaptation refers to ‘constructing a positive occupational identity and achieving occupational competence over time in the context of one’s environment’ (Kielhofner, 2008, p. 109).

In Theme 1, it was clear that participants faced the consequences of cancer and treatment on their occupational participation. Examples are evident in May’s reported fatigue and sensory issues and how they interfered with occupational participation. Hope’s voice affected her ability to teach. Rachel’s weakness and confidence affected her ability to dress and Elizabeth’s ability to walk. These findings were expected since evidence reports that occupations requiring the most physical, emotional and cognitive skills are usually most impacted by cancer treatment side effects (Fangel et al., 2013; Loubani et al., 2022). While these cancer treatment effects are well documented in the literature, our study also presented the role of the environment, including social groups of support and technology, to facilitate occupational participation of cancer survivors and, therefore, a way to support their occupational identity (Nizzero et al., 2017). Interestingly, although participants from our study reported participation with the support of others or assistive technology, sustained participation appeared to be insufficient when fatigue and other consequences of treatment were present, as identified in the discourses of Hope, May and Rachel.

In Theme 2, participants expressed dissatisfaction with not participating in occupations independently as in their past occupational participation. Although some theorists like Hammell (2014) had challenged that some cultural groups or communities value interdependence, our data demonstrated the opposite. Participants (Josephine, Elizabeth, Hope and Anna) expressed their needs for independence regarding several occupations such as work, cooking, engaging with friends and family. They reported their frustration (Elizabeth and Anna) and feeling useless (Josephine), not regarding their occupational identity, but to occupational competence to sustain a level of participation that reflected their occupational identity (Kielhofner, 2008).

The occupations referred to above by the participants illustrate how experiencing cancer affected their engagement in occupational roles as part of their identity. Moreover, the discourse reflects the participants comparing their occupational participation before the cancer experience, demonstrating how occupational competence was impacted, resulting in apparent problems of occupational adaptation (Kielhofner, 2008).

We could also identify possible cultural differences when comparing our results with the study of Israeli women in the chronic phase (Loubani et al., 2022). While participants from Israel released or ‘delegated authority’, accepting help from others, in our study, participants reported their difficulties in not being independent, which can be interpreted by Western values of independence and productivity, discussed by Hammel as part of Neoliberal ideas that influence societal values and expectations of roles (Hammell, 2014).

Hope reported that not being able to participate in occupations led her to feel ‘disabled’. Keesing et al. (2018) found that those after breast cancer often struggle with the conflicting identities they now have, such as being a patient to being a survivor. Not being able to participate in occupations may cause feelings of disability (Fangel et al., 2013). Nonetheless, our findings presented ambivalence amongst participants regarding an identity related to the condition they had; for example, Hope expressed acceptance of being disabled by treatment effects, while May and Rachel refused to be identified by a personal identity as a breast cancer survivor, indicating that their occupational identity remained despite difficulties in occupational participation. In light of the study of Palmadottir (2009), women who survive breast cancer can experience positive and negative perceptions that involve some contradictions as the ones identified in our study.

Individuals with a chronic illness are said to develop strategies to maintain their occupational participation and engagement (Maersk et al., 2021). Despite cancer treatment effects, Rachel, Hope, Josephine and May sustained their occupational participation using different means by asking for others to support, utilising technologies or strategies such as pacing their occupations, with breaks to allow them to rest, finding time allocated to sleep, adapting occupations (e.g., yoga), and identifying the right challenge according to their current skills, presence of fatigue, pain or lack of physicality.

These strategies can illustrate their occupational adaptation to accommodate an occupational competence, consistent with their occupational identities. Analogous findings were found in a longitudinal (3-year) qualitative study conducted by McGrath (2013) in Ireland. The author interviewed seven Irish women living with breast cancer who participated in a ten-session crochet class. The study identified that six participants developed lymphoedema following breast cancer treatment, requiring conservative treatment that was ignored due to their need to participate in occupational roles. Participants found self-management protocols for lymphoedema challenging to follow. They prioritised participation in familiar roles to support their occupational identity, despite challenges relating to vulnerability and risks of using their arms. For example, to hold and lift a child, two participants force their participation in roles as mother or grandmother, ignoring the risks to their arms, due to the strong connection with their occupational identity.

The discourse of participants in our study illustrates how their identities emerged through the interpretation of their lived experiences, for example, their thoughts and feelings about themselves are part of the concept of self, proposed by Christiansen (1999). Identity integrates life experiences, and the self is part of the identity construct since it includes thoughts, feelings and sensations (Christiansen, 1999). In our study, participants demonstrated their body awareness and a sense of who they are, to make choices and act. Our findings illuminate how participants cope with their condition and manage to participate in meaningful occupations. The lived experience reported by Rachel, Elizabeth, Hope and Josephine led to identifying some attributes related to the phenomenon of occupational engagement, such as a connection with the environment, body and mind, being present at the moment and a sense of entertainment (Black et al., 2019; Cruz et al., 2023).

Particularly, Hope described the involvement with the occupation of crafting, her knowledge of how being engaged in doing so shifted her focus on pain, in other words, the potential of involvement with an occupation in changing modes. At the same time, Rachel balanced her skills in the right challenge by reading a book. Our findings are consistent with a recent longitudinal study with women after a breast cancer treatment conducted in Israel, where participants expressed positive thinking and engagement in hobbies that shifted their focus away from breast cancer treatment and symptoms (Loubani et al., 2022). Additional evidence also reinforces the role of occupations in facilitating motivation and competence. In the qualitative study conducted with 18 women in different stages of cancer, including diagnosis, treatment and recovery, Palmadottir (2009) found that participants kept their routine of occupations as a strategy to ‘minds off’ the disease, increasing their feelings of being competent. Complementarily, in a study conducted in Denmark, all seven participants reported being too tired to engage in occupations; however, engaging in football fitness played a role in their motivation, where a participant with fatigue expressed that tiredness ‘disappeared’ when she started to practice (Thomsen et al, 2023).

Finally, it is known that receiving a cancer diagnosis requires an individual to reconstruct their narrative, making cancer a biographical disruption (Sleight and Clark, 2015). For those with cancer, the importance of engaging in mindful occupations has been discussed. This concept refers to occupations that take away thoughts and feelings from the biographical self and focus the attention of physical, emotional and cognitive resources on the current moment, therefore potentially increasing well-being (Sleight, 2017; Sleight and Clark, 2015). Goodman et al. (2019) found that mindful occupations can alleviate stress and anxiety for those with chronic illness as these occupations place less of an emphasis on ‘doing’ an occupation and more on being and becoming.

### Limitations

One potential limitation of our research is that the interviews were conducted virtually. This may have affected the rapport-building between the researcher and participants and the quality of the conversations. However, this was the most suitable method for conducting the interviews due to the COVID-19 pandemic and avoiding risks to participants since they may have had suppressed immune systems. Furthermore, the research was conducted in the UK with UK residents and a Western-centric perspective, resulting in possible transferability limitations to other perspectives, healthcare systems and countries.

The inclusion criteria of having received a diagnosis of breast cancer and treatment longer than a year possibly limited the number of participants. Although the interview questions comprised questions related to the illness and treatments, we recognise that variations in terms of time since diagnosis possibly limited the discussion of our findings. Because participants’ answers are drawn from retrieving past information about their experience post-diagnosis, this might be seen as a bias of inaccuracies in remembering details in depth. Nevertheless, the lived experience of participants shows rich information for future studies focused on the occupational identity of women after a breast cancer experience.

### Implications for occupational therapy

The evidence from the lived experience of participants indicates that there are occupational needs to be potentially addressed by occupational therapists in this population long after the acute phase of the illness. We emphasise the role occupational therapy could play in assisting those after a breast cancer diagnosis and treatment reconstructing and or sustaining occupational identity through the continuity of care. Since the evidence of interventions available with this population is predominantly bottom-up, such as physical interventions, exercises, cognitive or based on health management, and few studies are occupation-based (Molitor et al., 2023), occupation-centred interventions are needed. Our findings suggest that occupational adaptation is a human phenomenon not necessarily generated by an occupational process since participants were not under occupational therapy care. Yet, occupational therapy could facilitate the occupational adaptation process with this population, for example, by rebuilding identity and competence (Kielhofner, 2008) or discovering meaningful occupations that can generate new occupational identities, such as engaging in physical activity and its positive effects on occupational engagement and belonging (Thomsen et al., 2023).

## Conclusion

Our study described the lived experience of women after breast cancer and treatment regarding their occupational identity. Analysis of participants’ discourse revealed difficulties in participating in occupations that reinforced their occupational identities. Although the difficulties with participation were evident, our data suggests that occupational identity remained in the participants’ voices. Different strategies to sustain the participation that matched their occupational identities were identified, such as being present in the moment, knowing their limits of doing, resting and sleeping, and choosing occupations compatible with their skills. Future research can explore the potential of occupational engagement in alleviating the impact of cancer treatments on occupational identity, contributing to occupational adaptation outcomes.

**Key findings**

- Occupational identity remained in the participants’ discourse while the difficulty reported included independence in occupational participation.
- Occupational adaptation was expressed by the participants’ ability to employ strategies that matched their occupational identity.

**What the study has added**

After breast cancer and treatment, participants’ occupational identity was maintained despite challenges regarding competencies required to participate in occupational roles.

## Supplemental Material

sj-docx-1-bjo-10.1177_03080226231225103 – Supplemental material for An exploration into the occupational identity of women following breast cancer and treatment: A qualitative study Supplemental material, sj-docx-1-bjo-10.1177_03080226231225103 for An exploration into the occupational identity of women following breast cancer and treatment: A qualitative study by Courtney Hopkins, Angela Murphy, Rebecca Haythorne and Daniel Cezar da Cruz in British Journal of Occupational Therapy
