# Supplementary material for: An exploration into the occupational identity of women following breast cancer and treatment: A qualitative study
Source: Br J Occup Ther. 2024 Jan 9;87(6):383–91. doi: 10.1177/03080226231225103 (PMC12033690; doi:10.1177/03080226231225103)
Supplement: sj-docx-1-bjo-10.1177_03080226231225103 – Supplemental material for An exploration into the occupational identity of women following breast cancer and treatment: A qualitative study [file sj-docx-1-bjo-10.1177_03080226231225103.docx]

**Appendix 2**

**Glossary of Terms**

**Occupational participation**: A level of doing occurs closely within occupational roles (Bowyer et al., 2024). It refers to the broad doing occupations in a particular social and cultural context, such as Activities of Daily Living, work, and play (Kielhofner, 2008).

**Occupational roles**: incorporation of a social and personal “defined status and a related cluster of attitudes and actions” related to roles (Kielhofner, 2002, p. 78). Roles included expectations and/or “opportunities for occupational behavior, such as student, worker, volunteer, caregiver, home maintainer, friend, family member, religious participant, hobbyist/amateur, and participant in organizations” (Oakley et al, 1986, p.157).

**Occupational identity**: based on the idea that occupational participation supports building identity (Christiansen, 1999), Gary Kielhofner coined occupational identity as “a composite sense of who one is and wishes to become as an occupational being generated from one’s history of occupational participation” (Kielhofner, 2008, p. 106). Occupational identity is established by participation in roles, evolving across time in life, and is influenced by occupational competence (Bowyer et al., 2024).

**Occupational competence**: refers to the occupations a person engages in (Bowyer et al., 2024) or the “degree to which one sustains a pattern of occupational participation that reflects one’s occupational identity” (Kielhofner, 2008, p. 107). Occupational competence includes sustaining routines, occupational participation in a range of occupational roles with satisfaction and fulfilling role expectations, and having goals and actions according to personal values (Kielhofner, 2008).

**Occupational adaptation**: In the Model of Human Occupation (MOHO), Occupational adaptation is an internal process impacted by the environment and an outcome (Bowyer et al., 2024). Occupational adaptation is the longitudinal consequence of doing, described as the “construction of a positive occupational identity and achieving occupational competence over time in the context of one’s environment” (Kielhofner, 2008, p.107). Therefore, Occupational Adaptation has three components: identity, competence and the environmental impact (Bowyer et al., 2024).

**Mindful Occupation**: refers to the association of the concept of mindfulness applied to an occupation. Mindful occupation is described as occupations that benefit people “from greater presence and which they can use as formal meditation practices” (McVeigh, 2015, p.19). It is the engagement in everyday occupations that can “unlock the core self” to improve quality of life (Sleight, Clarke & Chan, 2015, p. 477). Mindful occupation can encompass occupational presence, awareness, engagement, well-being, and fulfilment (Goodman et al., 2019).

**Meaningful Occupation**: According to Christiansen and Haertl (2024) review, there is no consensus on the concept of meaningful occupation in occupational therapy literature, however, a synthesis of meaningful occupation comprises the attributes and features of an occupation: enjoyment, challenge, purpose consistent with self, choices, values, meaning in life and psychological needs, and satisfaction.

**Lived experience**: From a hermeneutic concept, lived experience represents the meaning attributed to the experience as a core to the concept; therefore, the lived experience cannot be only a description; it comprises “an interpretation of significance for the person” (Frechette et al., 2020, p. 3).

**References**

Bowyer P, Wolske J, Cabrera J and Fisher G (2024) Dimensions of Doing. In: Taylor, RR, Bowyer P and Fisher G (Eds). Kielhofner’s Model of Human Occupation. Sixth Edition, Philadelphia: Lippincott, pp.110-121.

Christiansen CH. (1999) Defining lives: Occupation as identity: An essay on competence, coherence, and the creation of meaning. American Journal of Occupational Therapy, 53(6), pp. 547–558. <https://doi.org/10.5014/ajot.53.6.547>

Christiansen C and Haertl K (2024) Essential Concepts of Occupation for Occupational Therapy: A Guide to Practice (1st ed.). New York: Routledge. <https://doi.org/10.4324/9781003242185>

Frechette J, Bitzas V, Aubry M, Kilpatrick K and Lavoie-Tremblay M (2020) Capturing Lived Experience: Methodological Considerations for Interpretive Phenomenological Inquiry. International Journal of Qualitative Methods, 19. <https://doi.org/10.1177/1609406920907254>

Goodman V, Wardrope B, Myers S, Cohen S, McCorquodale L and Kinsella EA (2019) Mindfulness and human occupation: A scoping review. Scandinavian Journal of Occupational Therapy, 26(3), pp.157-170. <https://doi.org/10.1080/11038128.2018.1483422>

Kielhofner G (2002) Habituation: patterns of daily occupation. In: Kielhofner G. Model of Human Occupation. 3^rd^ Edition, Philadelphia: Lippincott Willians & Wilkins, pp. 63-80.

Kielhofner G (2008) Dimensions of doing. In: Kielhofner G. Model of Human Occupation. 4^th^ Edition. Philadelphia: Lippincott Willians & Wilkins, pp.101-109.

McVeigh R (2015) Mindfulness in practice: developing an occupational therapy niche. Occupational Therapy Now, 17, pp.19–20.

Oakley F, Kielhofner G, Barris R, and Reichler RK (1986) The Role Checklist: Development and Empirical Assessment of Reliability. The Occupational Therapy Journal of Research, 6(3), pp.157-170. <https://doi.org/10.1177/153944928600600303>

Sleight A, Clark F and Chan TH (2015) Unlocking the Core Self: Mindful Occupation for Cancer Survivorship. Journal of Occupational Science, 22(4), pp.477-487. <https://doi.org/10.1080/14427591.2015.1008025>
